# Walking in pregnancy and prevention of insomnia in third trimester using pedometers: study protocol of Walking_Preg project (WPP). A randomized controlled trial

**DOI:** 10.1186/s12884-020-03225-y

**Published:** 2020-09-10

**Authors:** C. Amezcua-Prieto, M. Naveiro-Fuentes, N. Arco-Jiménez, R. Olmedo-Requena, R. Barrios-Rodríguez, I. Vico-Zúñiga, S. Manzanares Galán, J. Mozas-Moreno, J. J. Jiménez-Moleón, J. L. Gallo-Vallejo

**Affiliations:** 1 Department of Preventive Medicine and Public Health, Faculty of Medicine, University of Granada, Tower A, 8th Floor, Room 06, 18016 Granada, Spain; 2 Consortium for Biomedical Research in Epidemiology & Public Health (CIBER Epidemiología y Salud Pública-CIBERESP), 28029 Madrid, Spain; 3 Instituto de Investigación Biosanitaria ibs. GRANADA, Granada, Spain; 4 Obstetrics and Gynecology Service, Virgen de las Nieves University Hospital, 8014 Granada, Spain; 5 Department of Obstetrics and Gynecology, University of Granada, 18016 Granada, Spain

**Keywords:** Pedometer watch, Walking, Pregnancy, Insomnia

## Abstract

**Background:**

Previous studies in pregnancy have not focused in evaluating the effect of walking during pregnancy and prevention of insomnia. Our general objective is to determine the effect of a walking program in preventing the appearance of insomnia in the third trimester of pregnancy, increasing sleep quality and improving quality of life throughout pregnancy.

**Methods:**

Randomized Controlled trial in parallel in healthy sedentary pregnant women (*n* = 265), Walking_Preg Project (WPP), from university hospital in Granada, Spain. At 12th gestational week (GW), they will be invited to participate and randomly assigned to one of the three arms of study: the intervention group I1 (pedometer, goal of 11,000 steps/day), intervention group I2 (pedometer, no goal) and control (no pedometer). Duration of intervention: 13–32 GW. At 12th, 19th and 31st GW the average steps/day will be measured in groups I1 and I2. At 13th, 20th and 32nd GW, Athens Insomnia Scale (AIS), Pittsburgh Sleep Quality Index (PSQI), Adherence to Mediterranean Diet (AMD), physical activity (short IPAQ), quality of life (PSI), and consumption of toxic substances (caffeine, illegal drugs, alcohol and tobacco) will be collected. Student t test or Mann-Whitney U will be used to compare 19th and 31st GW mean of daily steps between I1 and I2 groups. To compare differences between groups in terms of frequency of insomnia/quality of life for each trimester of pregnancy, Pearson’s Chi-square test or Fisher’s exact test will be used. To determine differences in hours of sleep and quality of sleep throughout each trimester of pregnancy, analysis of variance or Friedman test will be used. McNemar-Bowker test will be used to assess differences in life quality in pre-post analyses in the 3 arms. We will use Stata 15 statistical software.

**Discussion:**

promoting walking in second half of pregnancy through use of pedometer and health pre-registration of a goal to be achieved –'10,000–11,000 steps a day’– should prevent appearance of insomnia in third trimester, will increase sleep quality and quality of life in pregnant women.

**Trial registration:**

ClinicalTrials.gov Identifier: NCT03735381. Registered 8th November, 2018.

## Background

The prevalence of insomnia at the beginning of pregnancy is around 40%, reaching over 60% in the third trimester of pregnancy [1–4]. Insomnia is associated with an increase of gestational diabetes mellitus (GDM), depression in pregnancy and postpartum, arterial hypertension and preeclampsia as a result of the increase in cytokine levels derived from sleep deprivation [5]. Insomnia is also involved in unplanned caesarean sections, prolonged labour, premature placental abruption, delayed intrauterine growth and prematurity [6].

It has been documented that practice of physical activity (PA) prevents the onset of insomnia in adults [7], postmenopausal women [8] and pregnant women [9]. In addition, the practice of regular PA (> 150 min/week) also increases the quality of sleep in adults [10] and in pregnancy [11]. However, only 20.3–30% of pregnant women are active [12, 13]. In fact, pregnant women spend more than 50% of their time in sedentary activities [14]. To make an objective assessment of this, a woman who counts less than 5000 steps/day on average could be considered sedentary [15].

Walking is one of the recommended activities in the guidelines of PA in pregnant women in Canada, Japan, Norway and Spain and the most carried out during pregnancy [16, 17]. However, the number of steps/days counted in the first trimester of pregnancy does not exceed 5000 steps [18], which is half of those recommended (10,000-11,000) [19] and decreases as the gestation progresses [18]. Walking during pregnancy regulates, prevents or delays the increase in blood pressure and cholesterol in healthy women [20], decreases the prevalence of type 2 diabetes mellitus [21] and improves glycaemic control of pregnant women with GDM [22]. Moreover, improves cardiorespiratory capacity [23], decreases stress and anxiety and improves sleep quality [24], increases quality of life [25] and reduces weight gain during pregnancy [26].

The pedometer is a cost-effective tool that increases the motivation to walk and increase the number of steps per day by 2000 to 2500 [27] compared to those who do not use it. The effectiveness of the pedometer is greater when establishing a goal to achieve [28]. Different studies carried out in pregnant women show that when all the participants of the study use a pedometer, the establishment of a goal of steps to be reached per day (10,000-11,000) increases the mood and number of steps walked by the person receiving such information [26, 29–33].

Some effects evaluated with the use of pedometers in pregnant women are the increase of mood [34], the decrease in weight gain in obese pregnant women [26, 30], the decrease in the incidence of preeclampsia [35] and the reduction of back pain [36]. However, there are no previous studies that analyse whether an intervention aimed at promoting PA in pregnant women, based on walking, helps to prevent insomnia that appears towards the end of pregnancy and how it could have an impact on the quality of life related to health and sleep quality as pregnancy progresses.

Our hypothesis is that a program to promote walking in the first half of pregnancy through the use of a pedometer and through the health pre-registration of a goal to be achieved –'10,000–11,000 steps a day’– prevents the appearance of insomnia in the third trimester of pregnancy, increases the quality of sleep and improves the quality of life of pregnant women.

### Aim

The main objective of this RCT is to determine the effect of a program aimed at promoting physical activity in pregnant women based on walking during pregnancy in the prevention of sleep hygiene disorders and improvement of the quality of life in the third trimester of pregnancy.

## Methods and design

**Fig. 1:**
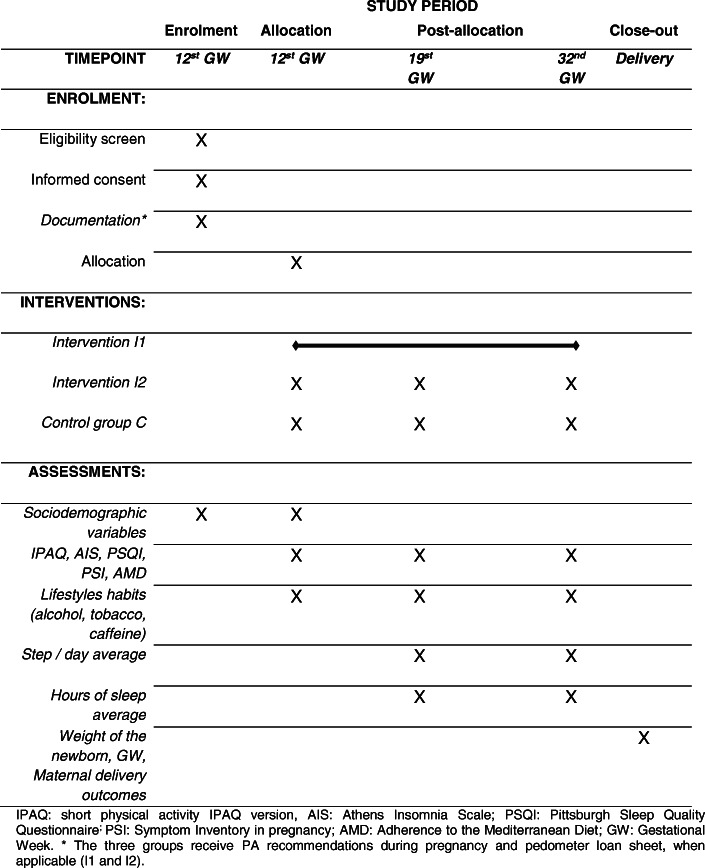
Walking_Preg Project (WPP) schedule of enrolment, interventions, and assessments

**Fig. 2:**
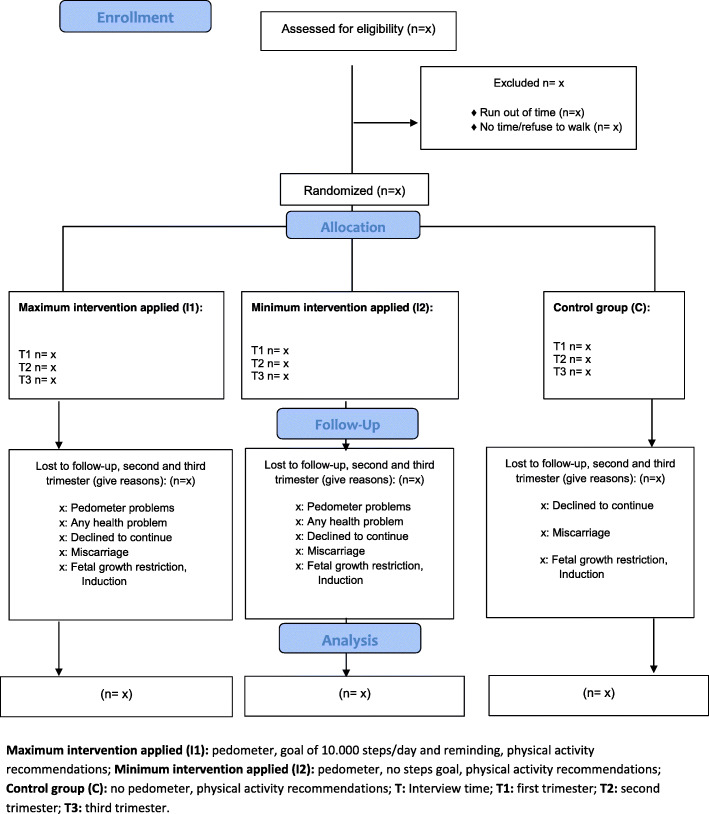
Walking_Preg Project (WPP) Consort Flow Diagram

The Walking_Preg Project, WPP study protocol follows the SPIRIT guidelines and the SPIRIT flow diagram is shown in Fig. 1. Walking_Preg Project (WPP) Consort Flow Diagram is presented in Fig. 2. This is a Randomized Controlled Trial with three groups in parallel: maximum intervention group (I1): with pedometer, information and objective of 10,000 steps/day to be achieved, minimum intervention group (I2), with pedometer and information but without goal of steps to be achieved, and control group (C), without pedometer but with information on physical activity recommendations and their benefits.

### Arms and interventions

The three groups will receive information about the recommendation of physical activity during pregnancy, according to ACOG guidelines [19].

#### Arm 1

Intervention 1 (I1), maximum intervention: pedometer + goal + reminds (maximum intervention women receive information about the goal of steps/day to be reached: 10,000-11,000. They will receive messages on the mobile phone remembering the goal to achieve and a notification that the researching staff will proceed to collect the average count of steps/day of the week prior to 20th and 32nd GW. Women will get recommendations on physical activity during pregnancy.

#### Arm 2

Intervention 2 (I2), minimum intervention: use of pedometer watch from 12th to 32nd GW (10 weeks). They will not receive goal information nor reminds to collect information from the pedometer. Women will be given recommendations on physical activity during pregnancy.

#### Arm 3

No Intervention, control group: without pedometer. Women will receive recommendations on physical activity during pregnancy.

### Selection criteria for eligibility

Inclusion Criteria: 1) Pregnant women with a minimum of 18 and maximum of 49 years of age; 2) Low medical risk pregnancies that will be attending in the Unit of Obstetrics and Gynecology, in a public third level Maternity Hospital in Granada (Spain); 3) Sedentary women (< 5 days/week of moderate-vigorous physical activity at least > 30 min.; equivalent to < 7000 steps/day); 4) With mobile phone and e-mail; 5) Without intellectual deficits or difficulty to understand the language.

Exclusion Criteria: 1) Chronic disease: diabetes, high-pressure, cardiac or respiratory disease, liver or kidney disease or mobility problems; 2) Women with relative or absolute repose needed; 3) Active women (> 5 days/week of moderate-vigorous physical activity at least > 30 min.; equivalent to > 7000 steps/day); 3) Insomnia at beginning of pregnancy or having drugs for sleep problems.

The study sample will consist of women who, meeting the inclusion criteria, agree to participate, upon request for voluntary collaboration and signing of two documents: 1) Informed consent approved by the Provincial Ethics Committee of Granada and 2) Pedometer loan certificate.

### Location and data collection

The collection of the study sample began in June 2019 in the Obstetrics and Gynecology Department, where the first uterine ultrasound is performed at 12th GW. The Service Management Unit of Obstetrics and Gynecology of the Maternal and Child Hospital –Virgen de las Nieves University Hospital Complex, in Granada– is the centre of care reference in the northern area of Granada, a third level hospital that serves a reference population of 439,035 corresponding to a sector of the Granada Metropolitan District and the Basic Health Zone of Alcalá la Real, Jaén. It has been a reference of the Northeast Health Management Area of Granada.

Walking_Preg Project (WPP) schedule of enrolment, interventions, and assessments is shown in Fig. 1. The health staff will inform each woman of the study by referring them to an adjoining consultation enabled for a member of the research team at the hospital. All women who meet the inclusion criteria and sign the informed consent and loan certificate will be invited to participate in the study to reach target sample size. During the pregnancy, three interviews will be conducted by training staff (12th, 20th and 32nd GW), collecting the information in an access database. The period of recruitment of pregnant women will end in December 2020. We have stopped the recruitment from March to August 2020, because of Covid-19. The period of follow-up of women in the study will take place from the time of recruitment of women (12th GW) until delivery.

### Sample size

First, we have carried out the theoretical calculation of the sample size for the definitive study (*n* = 265), based on the following premises: 1) Three study arms: *maximum intervention group*, pedometer + goal + recall (I1): minimum absolute reduction in the prevalence of low compliance with recommendations that are statistically significant to be detected, that it is 15% with respect to I1*; minimum intervention group*, pedometer (I2): minimum absolute reduction in the prevalence of low compliance with recommendations that are statistically significant to be detected, that is 30% with respect to C; and control group (C), with an estimated prevalence of low compliance with physical activity recommendations of 50% [12, 13]. 2) Alpha error: 5%; 3) Power of the study: 80%; 4) Comparison of I1 vs I2: from the previous premises, 120 women would be needed in each arm; Comparison of I1 vs C: from the previous premises, and taking as reference the 120 women of the I1 arm required for the previous comparison, 25 women in the C arm would be required.

A pilot of the previous design was carried out with 72 women not included in this study: 24 women in each arm of the study, interviewed twice (12th and 20th GW), during the months of February to May 2018. The recruitment period was 3 months. To select a total of 265 women, 1 year and 7 months would be required (considering holiday periods and Covid-19). We estimate 20–30% of losses in second and third interviews (due to neonatal loss or abandonment of the study).

### Randomization of participants and concealment

The Stata Ralloc program will generate a randomization sequence by blocks of *n* = 9 and 3 arms per block (A, B and C). In this way, each block of nine pregnant women treated consecutively will be assigned, in a random order, to the arms C (control), I1 (Intervention 1) and I2 (Intervention 2): *C I1 I1 I1 I1 I2 I2 I2 I2*, up to complete the assignment of the 265 pregnant women. The sequence will be generated by the data analysts and kept in the monitoring centre, remaining at all times hidden to those clinicians responsible for the enrolment of pregnant women. Allocation sequence will be concealed until women are assigned to interventions by the research staff/interviewer. This way, the follow-up centre will be contacted each time the interviewer has a pregnant woman, so that they are informed of the arm to which she will be assigned.

There will be concealment for the people in charge of carrying out the evaluation and analysis of the data because they will manage the database without personal women information or knowledge about the group intervention, where women will be allocated. Due to the nature of the study, neither the participants nor the professionals who indicate the intervention can be blinded. Participants will know they will be selected for either a pedometer group or a non-pedometer group. They will not know if the intervention is minimum (pedometer only) or maximum (pedometer and a goal of 11,000 steps/day).

### Instruments

These are the main sources of information:

1) **Structured interview** that will be conducted in person at 13th, 20th and 32nd GW. The duration of the interviews will take approximately 35–40 min, which includes: the Athens Insomnia Scale (in English, AIS-8) [37], the Pittsburgh Sleep Quality Questionnaire [38], the Symptom Inventory in pregnancy [39, 40], short IPAQ [41], Adherence to the Mediterranean Diet, with Predimed questionnaire without wine [42]; 2) **Record of the average steps/day** of weeks 19 and 31 of gestation (information downloaded from the pedometer) and quantification of average hours of sleep at 19th and 31st GW (information downloaded from the Xiaomi Mi Band 2™ pedometer) (Fig. 1); 3) **Clinical history:** to collect the variables related to birth (tertiary outcome variables) and 4) Subsequent **telephone contact** if necessary (correction of missing data).

### Baseline variables collected

Variables collected only in the first interview (13th GW) will be: *Sociodemographic variables* –age, number of previous children, number of children under 3 years, number of previous abortions, Spanish or foreign nationality, stable couple, level of studies, occupation of woman and partner (from these last two variables and taking into account the highest value, social class was calculated, with five categories in descending order I, II, III, IV and V, according to the Spanish Society of Epidemiology [43]); *Anthropometric variables:* height and pre-pregnancy weight.

### Variables collected in all interviews

- Diagnosis of pathologies in pregnancy: gestational diabetes, high blood pressure induced by pregnancy or preeclampsia and eclampsia.

- Drug use:

1) Consumption of **illegal drugs**: use of opiates, cocaine, cannabis and other drugs (specifying which) is recorded and if so, if the consumption is daily (number of times), weekly, monthly or occasional (number of days of consumption per week, per month or occasional and number of times per day of consumption); 2) Consumption of **medication and supplements** prescribed or not by a physician, reason and duration; 3) **Smoking habit**: 1. Active daily smoker; 2. Occasional smoker; 3. Recent former smoker (< 6 months); 4. Former smoker (> 6 months); 5. Never smoked [44]. *Active smoker*: average number of cigarettes per day; age of onset of consumption; *Former smoker:* How long ago did you stop smoking? Years or months. *Passive tobacco:* does your partner regularly smoke at home? If so: cigarettes/day; 4) **Alcohol:** number of drinks consumed daily, weekly, monthly or occasionally (wine or cava, beer with alcohol or cider, high-grade alcohol) [44] and 5) **Caffeine** consumption: number of cups (or bottles) that are consumed daily, weekly, monthly or occasionally of each of the following beverages: coffee, decaffeinated coffee, tea, cola, energy drinks and chocolates (milk or non-milk chocolate tablet).

*-* Physical activity*:* short IPAQ questionnaire [41], which includes the weekly frequency and minutes of completion of intense, moderate and walking physical activity. It allows the study sample to be classified into three categories according to their activity per week: low level or inactive (category I), moderate (category II) and active (category III).

*-* Diet*:* Adherence to the Mediterranean Diet pattern questionnaire, without wine [42]; 13-item questionnaire (olive oil, vegetables, fruits, red meat, chicken, fish, legumes, butter/margarine, carbonated drinks, industrial pastries, nuts, stir-fry) that are part of the Mediterranean diet. The higher the score obtained on the scale, the greater the adherence to the Mediterranean diet and is valued as acceptable adherence from 8 points.

### Outcome measures

Primary outcome measures in the three arms:

1) ***Prevalence of insomnia*** in third trimester of pregnancy (time frame: 32nd GW) will be evaluated with the Insomnia Athens Scale (IAS) [37], an eight-item scale that assesses the quantity and quality of sleep (first five items) and the diurnal repercussion of insomnia (daytime sleepiness, physical and mental functioning and well-being during the day). Each of the items scores from 0 to 3 (affectation of the item from light to severe). The total score ranges from 0 to 24 points, considering insomnia from a score equal to or greater than 6 points (Sensitivity = 93% and Specificity = 85%) or equal to or greater than 7 points (Sensitivity = 84% and Specificity = 90%).

2) ***Change in mean steps/day*** after intervention in the arms using pedometer register (time frame: 19th and 32nd GW).

Secondary outcome measures in the three arms (time frame: 12th, 19th and 32nd GW): ***Quality of sleep*** of pregnant women measure with the Pittsburgh Questionnaire (PSQI) [38], it assesses the quality of the individual’s sleep during the previous month. It consists of 19 items grouped into seven components: quality, latency, duration, efficiency and sleep disturbances, use of medication for sleep and diurnal dysfunction; each component is scored from 0 to 3, according to a Likert scale in which zero is the absence of the symptom and 3 is its maximum presence. This will result in a score that ranges from 0 to 21 points (the higher score, the worse sleep quality): a PSQI score of > 5 indicates poor sleep quality, with a diagnostic sensitivity of 89.6% and a specificity of 86.5%.

Other pre-specified outcome measures in the three arms:

1. ***Quality of life*** of women through pregnancy (time frame: 12th, 19th and 32nd GW), measure with the Pregnancy Symptom Inventory (PSI) [39, 40]: 41-item Likert scale developed from a group of experts and focus groups. It registers the range of symptoms that appears in the last month (Likert scale from 0 to 4: never, rarely, sometimes, often) and its impact on quality of life, by affecting the activities of daily life (scale Likert from 0 to 3: it does not limit me, it limits me a little, it limits me a lot). It has been adapted to Spanish pregnant women and is reliable (Kappa coefficient range = 0.6–0.9) [40]; 2. ***Women weight gain*** in kilograms (time frame: 12th and 32nd GW), measured with the hospital scale; 3. ***Weight of the new-born*** (time frame: delivery) collected from the clinical history of the women; 4. ***Gestational week at delivery*** (time frame: delivery) collected from the clinical history of the women.

### Data analysis

A descriptive analysis of the variables collected in the study will be carried out. Measures of central tendency and dispersion will be calculated for the numerical variables and absolute and relative frequencies for the qualitative ones. The normality of the variables will be checked with the Kolmogorov-Smirnov test, in order to determine whether parametric or non-parametric tests will be carried out later. 95% confidence intervals (CI) will be obtained for both means and proportions.

For the contrast of the hypothesis of equality between the group of patients I1 and the group of patients I2, in terms of the number of daily steps and for each GW (19th and 31st), the Student t test will be used for independent samples or Mann-Whitney U, as appropriate. Subsequently, to assess whether there are differences between the groups with respect to the frequency of insomnia/quality of life for each trimester of pregnancy, the Pearson’s Chi-square test or Fisher’s exact test will be used when necessary.

On the other hand, to determine if differences are found between the quantitative variables (hours of sleep, quality of sleep) throughout the study period (first, second and third trimester) among the participants, the analysis of variance will be used for measurements repeated in the parametric case. In the case of having to use a non-parametric test, the Friedman test will be used. Subsequently, to assess the differences in the quality of life, for each of the groups, before starting the walking program and after finishing it (pre-post), the McNemar-Bowker test will be used.

For the contrast of the hypothesis of equality between the three groups of patients, regarding the weight gained during pregnancy (until 34 ^st^ GW), the weight of the newborn and the gestational week, an analysis of the variance or the test of Kruskall-Wallis will be used, as appropriate. In case of statistical significance, multiple comparisons will be obtained using the Student’s parametric test or the non-parametric Mann-Whitney U test applying Bonferroni correction.

Finally, a bivariate mixed-effects logistic regression model will be carried out to determine which variables influence the greater frequency of insomnia, considering the latter quantitatively. To delimit the extent to which the associations that appear are explainable by the effect of the rest of the variables included in the study, a multivariate mixed-effects logistic regression model will be constructed. 95% confidence intervals (CI) will be obtained. All contrasts will be bilateral and statistical significance will be considered when the *p* value is ≤0.05. The data analysis will be carried out by intention to process and by protocol, using the Stata / SE v15.0 statistical package.

## Discussion

Currently, about 60% of pregnant women have insomnia towards the end of pregnancy [1–4]. This study aims to reduce the prevalence of insomnia in the third trimester of pregnancy by 33% in the pedometer + goal group (maximum intervention) and, on the other hand, to reduce the prevalence of insomnia by 16.6% in the pedometer-only group (minimum intervention), compared to the control group (usual advice without pedometer).

Women in the maximum intervention group are expected to have 33% less insomnia in the third trimester of gestation compared to pregnant women in the control group. Furthermore, in the intervention groups, sleep quality will improve (Pittsburgh score < 5) [38], the frequency of symptoms associated with pregnancy (asthenia, anxiety, cramps) will decrease and the performance of daily life activities (measured with the PSI questionnaire) will improve, compared to the control group.

The intervention requires health professional to explain the use of the pedometer and establish a therapeutic alliance with the pregnant woman to reach a goal. The benefits are expected to be obtained during the project and after its completion: 1) During the project, since the participating women will increase their motivation to walk, due to the use of the pedometer, and will benefit from the inclusion of PA in their daily life, such as decreased sedentary lifestyle and weight control in pregnancy. This will even encourage the practice of walking from their closest social or family bond: 2) In the short term, we will know if the expected potential change has managed to reduce the prevalence of insomnia in the third trimester of pregnancy, by at least 33%.

Direct and indirect costs are expected to be saved: 1) Direct cost in pregnant women enrolled in the study —decreasing sleep-related disorders, increasing quality of sleep, quality of life and general well-being; 2) Indirect costs for the Andalusian Health Service: reduction in the number of visits in Primary Health Care derived from the direct consequences of walking during pregnancy, cost reduction in drugs related to the discomfort of pregnancy. Depression, pregnancy weight control, pregnancy-induced high blood pressure, or back pain will be mitigated by promoting pregnancy walking.

This study will set support for the elaboration of health promotion protocols in pregnant women through walking. The findings of the beneficial effect derived from the research can be incorporated into the usual advice of clinical practice. The results should be echoed among health professionals involved in the care process of pregnancy, childbirth and the puerperium, as well as the scientific community.

Finally, this study will encourage walking in pregnancy. In return, the participants will increase their general well-being by improving their quality of life and sleep.

## Data Availability

No results or data are available to share.

## Abbreviations

WPP: Walking_Preg Project
GW: Gestational Weight
AIS: Athens Insomnia Scale
PSQI: Pittsburgh Sleep Quality Index
AMD: Adherence to Mediterranean Diet
IPAQ: International Physical Activity Questionnaire
PSI: Pregnancy Symptom Inventory
GDM: Gestational Diabetes Mellitus
PA: Physical Activity
RCT: Randomized Controlled Trial
ACOG: American College of Obstetrics and Gynaecology
